# Genetic diversity within *Mycobacterium tuberculosis* complex impacts on the accuracy of genotypic pyrazinamide drug-susceptibility assay

**DOI:** 10.1016/j.tube.2014.04.002

**Published:** 2014-07

**Authors:** Claudio U. Köser, Iñaki Comas, Silke Feuerriegel, Stefan Niemann, Sebastien Gagneux, Sharon J. Peacock

**Affiliations:** Department of Medicine, University of Cambridge, Cambridge, United Kingdom; Genomics and Health Unit, FISABIO, Valencia, Spain; CIBER (Centros de Investigación Biomédica en Red) in Epidemiology and Public Health, Spain; Molecular Mycobacteriology, Borstel, Germany; German Centre for Infection Research, Research Centre Borstel, Borstel, Germany; Department of Medical Parasitology and Infection Biology, Swiss Tropical and Public Health Institute, Basel, Switzerland; University of Basel, Basel, Switzerland; Department of Medicine, University of Cambridge, Cambridge, United Kingdom; Clinical Microbiology and Public Health Laboratory, Public Health England, Cambridge, United Kingdom; Cambridge University Hospitals NHS Foundation Trust, Cambridge, United Kingdom; Wellcome Trust Sanger Institute, Wellcome Trust Genome Campus, Hinxton, United Kingdom

**Keywords:** *Mycobacterium tuberculosis* complex, Pyrazinamide resistance, Phylogenetic diversity

To the Editor,

We agree with Pholwat et al. [1] that fast genotypic methods will play an increasingly prominent role in drug-susceptibility testing (DST) for the *Mycobacterium tuberculosis* complex (MTBC) [2]. They reported on the evaluation of a high-resolution melt assay (HMR) to detect pyrazinamide (PZA) resistance, which has the advantage of speed and simplicity. Two of 96 test isolates were PZA susceptible but contained a synonymous *pncA* (*Rv2043c*) tcC/tcT Ser65Ser mutation and were falsely classified as resistant by HMR. The isolates in this study came almost exclusively from two countries (Thailand and Tanzania), and we questioned how disseminated this mutation was in the MTBC population at large. A literature review suggested that this silent mutation is largely specific to the Central Asian (CAS) genotype, although not all CAS strains harbour this polymorphism [3], [4], [5], [6], [7], [8], [9], [10]. We also sought the mutation in a globally representative collection of MTBC genomes (*n* = 219), which confirmed these findings (Figure 1) [11]. 83% of the isolates of the East African-Indian lineage 3, which encompasses the CAS genotype [12], shared the mutation in question, whereas the 6 most phylogenetically basal lineage 3 isolates lacked the polymorphism. These isolates were not easily identifiable using spoligotyping alone as they included a number of Shared International Types (SITs): SIT1 (pseudo-Beijing [13], [14]), SIT26, SIT486 and SIT1200. In fact, SIT26, the most frequent CAS spoligotype globally [15], was paraphyletic (i.e. it included isolates with and without the synonymous mutation (Supplemental Figure 1)).

**Figure 1:**
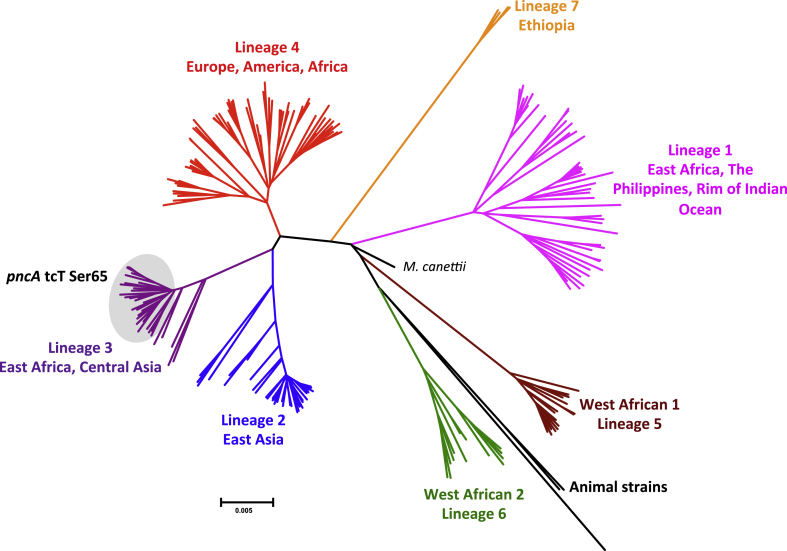
Whole-genome phylogeny of 219 isolates of all major MTBC lineages [11]. Only the more phylogenetically recent lineage 3 isolates shared the *pncA* tcT variant at Ser65, whereas the 6 remaining lineage 3 isolates and all isolates from other lineages had the tcC variant (including the lineage 4 *M. tuberculosis* H37Rv laboratory strain that is used as the reference/wild-type sequence for sequence analyses). The spoligotypes for the lineage 3 isolates can be found in Supplemental Figure 1.

The data by Pholwat et al. are in agreement with our findings as they found this synonymous mutation in 7/15 (47%) of Tanzanian isolates, where lineage 3 strains are known to be dominant [12], [16], and in none of the isolates from Thailand or the United States, where lineage 3 strains are rare [12], [17]. As a result, the specificity of their HMR assay depends on the local MTBC population structure (i.e. in countries in which lineage 3 isolates are widespread the number of false-resistant results will likely exceed the number of true-positives). By contrast, the Nipro Corporation avoided this flaw by including an additional probe to compensate for the Ser65Ser polymorphism when designing their *pncA* line probe assay [18], [19]. This underlines that the MTBC diversity has to be considered when designing and validating genotypic DST assays [19], [20].

## Funding

This work was supported by a grant from the Department of Health, Wellcome Trust and the Health Innovation Challenge Fund (HICF-T5-342 and WT098600) and Public Health England (to S.J.P.). C.U.K. is a Junior Research Fellow at Wolfson College, Cambridge. I.C. is supported by the Spanish Government (Ramón y Cajal programme RYC-2012-10627).

## Disclaimer

This publication presents independent research supported by the Health Innovation Challenge Fund (HICF-T5-342 and WT098600), a parallel funding partnership between the Department of Health and Wellcome Trust. The views expressed in this publication are those of the authors and not necessarily those of the Department of Health or Wellcome Trust.

## Transparency declarations

S.J.P. is a consultant for Pfizer Inc and received funding for travel and accommodation from Illumina Inc.
