# Supplementary material for: Genetic diversity within Mycobacterium tuberculosis complex impacts on the accuracy of genotypic pyrazinamide drug-susceptibility assay
Source: Tuberculosis (Edinb). 2014 Jul;94(4):451–3. doi: 10.1016/j.tube.2014.04.002 (PMC4075347; doi:10.1016/j.tube.2014.04.002)

Supplement for “Genetic diversity within *Mycobacterium tuberculosis* complex impacts on the accuracy of genotypic pyrazinamide drug-susceptibility assay”

Supplemental Figure 1

Detailed genome-based phylogeny of lineage 3 isolates from Figure 1 with the associated spoligotypes, where available.

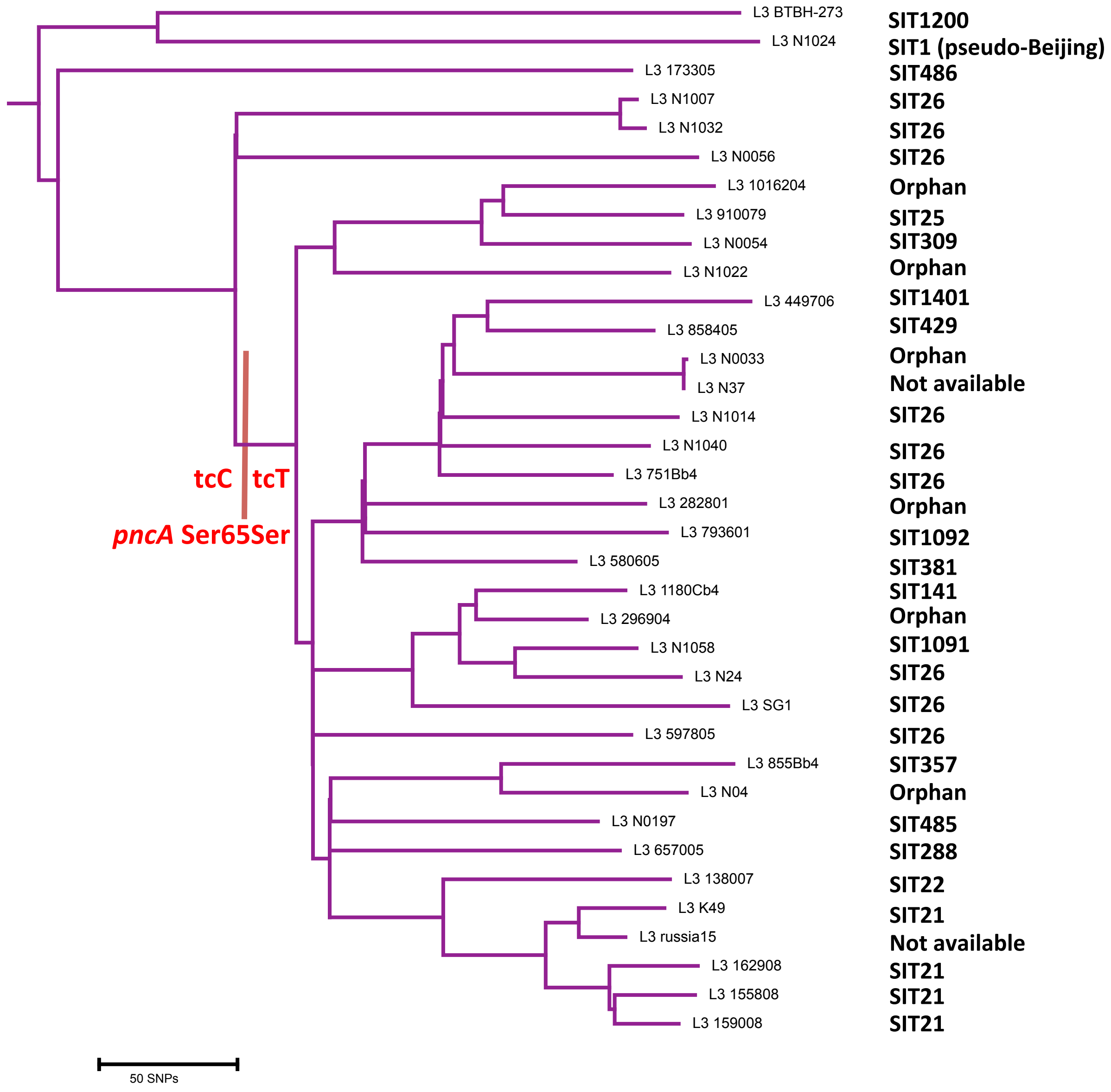

Supplement: Supplementary file 1 [file mmc1.pdf]
